# *Notes from the Field:* Recreational Nitrous Oxide Misuse — Michigan, 2019–2023

**DOI:** 10.15585/mmwr.mm7412a3

**Published:** 2025-04-10

**Authors:** Varun Vohra, Hannah Matthews, Gabrielle Stroh-Steiner

**Affiliations:** ^1^Michigan Poison & Drug Information Center, Department of Emergency Medicine, Wayne State University School of Medicine, Detroit, Michigan; ^2^Michigan Department of Health and Human Services.

**Summary**

**What is already known about this topic?**

Nitrous oxide is a widely accessible recreational substance that induces rapid euphoric and hallucinogenic effects. Often thought by users to be harmless, nitrous oxide can cause severe neurologic, cardiovascular, and psychiatric signs and symptoms with repeated use.

**What is added by this report?**

In Michigan in 2023, annual poison center exposures, emergency department visits, and emergency medical service responses related to nitrous oxide misuse were four to five times those in 2019.

**What are the implications for public health practice?**

Widespread availability of nitrous oxide and increasing medical encounters related to its use warrant targeted public health education for recreational users, parents, caregivers, and clinicians. Because no reliable screening test for nitrous oxide is available, increased clinical awareness, including a detailed recreational drug history, is needed for accurate and timely diagnosis of misuse.

Nitrous oxide is a colorless gas used clinically as an inhalational anesthetic, analgesic, and anxiolytic. It is a common component of some commercial products, most notably as a propellant in steel aerosol containers used in whipped cream dispensers, from which it can be inhaled (whippets) (*1*–*3*). Acute nitrous oxide use induces a rapid onset of euphoric, anxiolytic, and hallucinogenic effects that are short-lived, disappearing within minutes (*2*). The inhalant is an increasingly popular recreational substance, particularly among teenagers and young adults (aged 20–39 years), offering users a low cost and currently legal option easily accessible online and widely available at vape stores, grocery and convenience stores, and gas stations (*1*–*3*). Despite misconceptions among recreational users that nitrous oxide is safe (*1*,*2*), frequent or chronic long-term use can cause disabling neurologic sequelae, including neuronal demyelination and subacute combined spinal cord degeneration consequent to functional vitamin B12 deficiency (*2*,*4*). Treatment involves permanent cessation of nitrous oxide use along with high-dose intramuscular vitamin B12 supplementation; recovery is often protracted and incomplete (*4*,*5*).

In late 2023, an increase in the number of patients hospitalized with neurologic signs and symptoms secondary to nitrous oxide misuse was detected in Michigan Poison & Drug Information Center (MiPDC) data by the MiPDC director and clinical toxicologist. Toxicosurveillance monitoring was prompted by an observed corresponding increase in nitrous oxide–associated poisoning consultations involving the poison center and its toxicologists. MiPDC and the Michigan Department of Health and Human Services collaborated to investigate poison center cases, emergency department (ED) visits, and emergency medical service (EMS) responses to analyze trends in nitrous oxide–associated poisoning exposures and health care encounters in Michigan.

## Investigation and Outcomes

Poison center data from January 1, 2019, to December 31, 2023, were accessed by the poison center director and clinical toxicologist via the MiPDC ToxSentry electronic medical record database. Michigan ED visits (via Michigan Syndromic Surveillance System) and EMS responses (via biospatial, Inc.) were analyzed for key words (nitrous, whippets, and spelling variations) and *International Classification of Diseases, Tenth Revision, Clinical Modification* diagnosis code T59.0 (toxic effects of nitrogen oxides). Two epidemiologists and a clinical toxicologist reviewed EMS and ED cases, removing 59 of 383 that did not indicate nitrous oxide misuse. This research was performed in accordance with ethical principles, including ensuring responsible data handling and maintaining confidentiality. This surveillance research study was reviewed and approved as exempt human subjects research by the Institutional Review Board associated with MiPDC as well as falling under the ongoing public health surveillance responsibilities from the Michigan Department of Health and Human Services.

During 2019–2023, 144 poison center cases, 132 ED visits, and 192 EMS responses involving nitrous oxide were identified. Notable increases were observed in 2023 as compared with 2019 in poison center calls (10 to 48), ED visits (seven to 60), and EMS responses (15 to 78) (Figure). Nitrous oxide events occurred most frequently among persons aged 20–39 years (median age: poison center = 26 [IQR 21–33]; ED = 29 [IQR 24–33]; and EMS = 32 [IQR 25–39] years) and in metropolitan counties (poison center = 91.7%; ED = 94.7%; and EMS = 94.3% of events). Among the 192 EMS responses, 14 (7.3%) involved fatalities, including three suspected suicides. Cause of death cannot be determined in EMS data, but nitrous oxide involvement was documented among these fatalities. Polysubstance involvement occurred in 30% of poison center cases, most commonly benzodiazepines (9.7%), cannabis/delta-9-tetrahydrocannabinol (9.0%), and alcohol (8.3%). The most common clinical effects among nitrous oxide–involved poison center cases were tachycardia (19.4%), other-neurologic (18.8%), and numbness (16.7%) (Supplementary Table, https://stacks.cdc.gov/view/cdc/177219#tabs-3).

**FIGURE:**
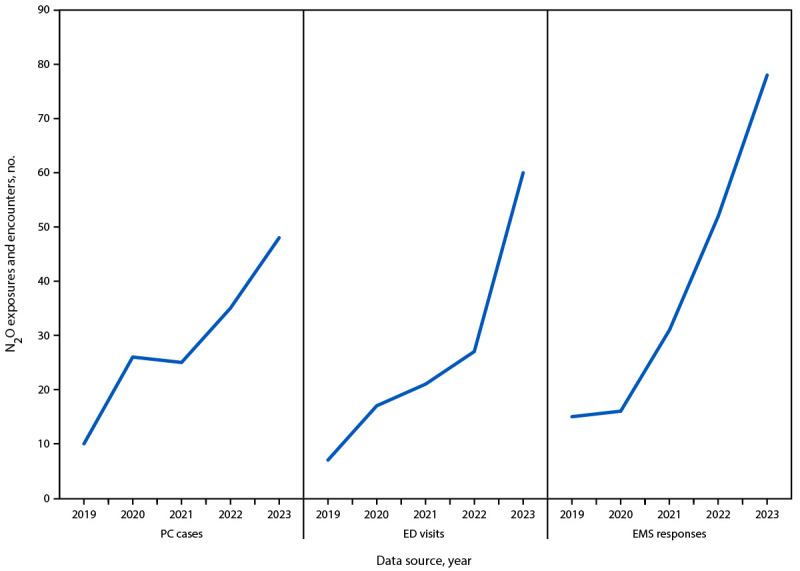
Poison center cases, emergency department visits, and emergency medical service responses related to recreational nitrous oxide misuse — Michigan, 2019–2023 **Abbreviations:** ED = emergency department; EMS = emergency medical service; N_2_O = nitrous oxide; PC = poison center.

## Preliminary Conclusions and Actions

These data demonstrate a sharp increase in adverse medical encounters associated with nitrous oxide misuse in Michigan during 2019–2023. Widespread availability, ease of access, and minimal legislative restrictions are potential factors contributing to the observed increase (*1*,*3*,*4*). These statewide data likely underestimate actual morbidity and mortality, because clinical suspicion of nitrous oxide toxicity is often low (*1*,*2*). No reliable screening test for nitrous oxide is available (*1*,*2*). Therefore, diagnosis is nuanced and predicated upon obtaining a detailed recreational drug history and interpreting surrogate serum biomarkers in the context of patient diagnostics and symptomatology (*2*,*4*,*5*). Toxicity stemming from chronic long-term nitrous oxide misuse is primarily characterized by neurologic, psychiatric, and hematologic findings (*2*,*4*,*5*). Neurologic manifestations include weakness, gait instability, and paresthesia that can progress to sensorimotor polyneuropathy with demyelinating features with or without evidence of subacute combined spinal cord degeneration (*2*,*4*). Psychiatric symptoms can include hallucinations, anxiety, depression, delirium, and memory impairment (*2*,*5*). Chronic nitrous oxide misuse–related hematologic abnormalities include those resembling megaloblastic or pernicious anemias, and myelosuppression (*2*). Recent evidence suggests a direct or indirect pathophysiological role in pulmonary embolism and deep vein thrombosis (*2*).

Although nitrous oxide is not a federally controlled substance (*3*), individual states, including Michigan, have enacted or are considering expanded legislation to regulate nitrous oxide possession, sale, and distribution. This report supports the need for enhanced surveillance of nitrous oxide exposures using poison center and health care encounter data. Targeted education for recreational users, parents, caregivers, and clinicians in conjunction with prevention campaigns to warn the public of the dangers of nitrous oxide misuse are warranted.
